# Two Natural Alkaloids Synergistically Induce Apoptosis in Breast Cancer Cells by Inhibiting STAT3 Activation

**DOI:** 10.3390/molecules25010216

**Published:** 2020-01-05

**Authors:** Di Chen, Yangmin Ma, Zhiyu Guo, Li Liu, Yaru Yang, Yuru Wang, Bonan Pan, Luyang Wu, Yuyu Hui, Wenjuan Yang

**Affiliations:** 1 Shaanxi Key Laboratory of Chemical Additives for Industry, Shaanxi University of Science and Technology, Xi’an 710021, China; observercd@163.com (D.C.); wuluyang@sust.edu.cn (L.W.); 18392995152@163.com (Y.H.); yangwenjuan@sust.edu.cn (W.Y.); 2 School of Food and Biological Engineering, Shaanxi University of Science and Technology, Xi’an 710021, China; 3 Institute of Basic Medical Sciences, Xi’an Medical University, Xi’an 710021, China; gzy_xyu@163.com (Z.G.); ll_2508@163.com (L.L.); yyr_xhu@163.com (Y.Y.); wangyuru_xyu@163.com (Y.W.); pbn5333@163.com (B.P.)

**Keywords:** piperine, piperlongumine, breast cancer, apoptosis, STAT3

## Abstract

Breast cancer has become a worldwide threat, and chemotherapy remains a routine treatment. Patients are forced to receive continuous chemotherapy and suffer from severe side effects and poor prognosis. Natural alkaloids, such as piperine (PP) and piperlongumine (PL), are expected to become a new strategy against breast cancer due to their reliable anticancer potential. In the present study, cell viability, flow cytometry, and Western blot assays were performed to evaluate the suppression effect of PP and PL, alone or in combination. Data showed that PP and PL synergistically inhibited breast cancer cells proliferation at lower doses, while only weak killing effect was observed in normal breast cells, indicating a good selectivity. Furthermore, apoptosis and STAT3 signaling pathway-associated protein levels were analyzed. We demonstrated that PP and PL in combination inhibit STAT3 phosphorylation and regulate downstream molecules to induce apoptosis in breast cancer cells. Taken together, these results revealed that inactivation of STAT3 was a novel mechanism with treatment of PP and PL, suggesting that combination application of natural alkaloids may be a potential strategy for prevention and therapy of breast cancer.

## 1. Introduction

Breast cancer has become a serious threat to women worldwide. Although there have been approved drugs targeted at hormone receptors, including the estrogen receptor (ER), progesterone receptor (PR), and human epidermal growth factor receptor-2 (HER2) [1], chemotherapy remains a routine treatment for breast cancer, particularly for triple-negative breast cancer (TNBC), which lacks major effective targets [2]. Unfortunately, nonselective killing and drug resistance to chemotherapeutic agents have become obstacles in current therapy, resulting in severe side effects and poor prognosis. Therefore, developing reliable and less toxic anticancer agents from natural sources has become a new approach [3].

Piperine (PP) is a major plant alkaloid isolated from black pepper (*Piper nigrum* L.) and long pepper (*Piper longum* L.) [4], which have been used in food or traditional medicine worldwide. Piperine exhibits a variety of pharmacological properties, including acting as an anticonvulsant [5], an antioxidant [6], an anti-inflammatory [7], an anti-angiogenic [8], an anti-bacterial, and an anticancer compound. Recent studies have reported that piperine can be cytotoxic to multiple animal and human cancer cells, such as 4T1 mouse breast cancer cells [9], PC-3 human prostate cancer cells [10], and A2780 human ovarian cancer cells [11]. Moreover, PP affects diverse signaling pathways associated with cancer cell growth and survival, including mitogen-activated protein kinase (MAPK), PI3K/Akt, and STAT3 pathways [12,13]. PP regulation of the above signaling pathways causes cell cycle arrest and apoptosis, eventually leading to cancer cell death. These findings suggest that PP may have potential as a therapeutic agent for the prevention and treatment of breast cancer.

Piperlongumine (PL) is another natural alkaloid first isolated from *Piper longum* L. in the 1960s. Previous studies identified PL as a potent anticancer compound with reliable selectivity [14]. The killing effects of PL involve inhibiting proliferation [15], inducing apoptosis [16], promoting ROS production [17], inhibiting migration and invasion [18], as well as sensitizing other chemotherapy agents [19,20], which occurs regardless of p53 status [21]. In addition, multiple signaling pathways are activated or inactivated by PL, including MAPK [22], PI3K/Akt/mTOR [16], nuclear factor kappa B (NF-κB) [23], GSTP1 [24], and TrxR1 [25]. Besides, PL has been confirmed to be a natural inhibitor of STAT3. However, problems such as extremely low natural content, complex synthesis route, organ toxicity [14], and poor solubility [26] at higher doses limit the prospect of PL as an anticancer drug. Nevertheless, PL is worth further research due to its good selectivity and ability to sensitize cells to other agents [27,28].

Monotherapy often leads to tumor recurrence and drug resistance [29], while combination therapy has become a novel and promising approach in current cancer treatment [30,31]. Although both PP and PL have wide anticancer potential, their deficiencies make it difficult to fight cancer alone. Considering the same isolation source and similar killing mechanisms, we intend to evaluate whether these two natural alkaloids together show better anticancer potential. In the present study, we examined the effects of PP and PL alone or in combination on cell proliferation and apoptosis in breast cancer and normal cells. MTT and flow cytometry data demonstrated that PP and PL showed more potent anticancer potential with better selectivity with combination treatment. Signaling pathway studies demonstrated that PP and PL inhibit STAT3 activation and regulate apoptosis-related proteins in breast cancer cells. These findings provide theoretical evidence for the future use of natural alkaloids for breast cancer prevention and therapy.

## 2. Results

### 2.1. Piperine and Piperlongumine Inhibit the Proliferation of Breast Cancer Cells and Normal Breast Cells

**Figure 1:**
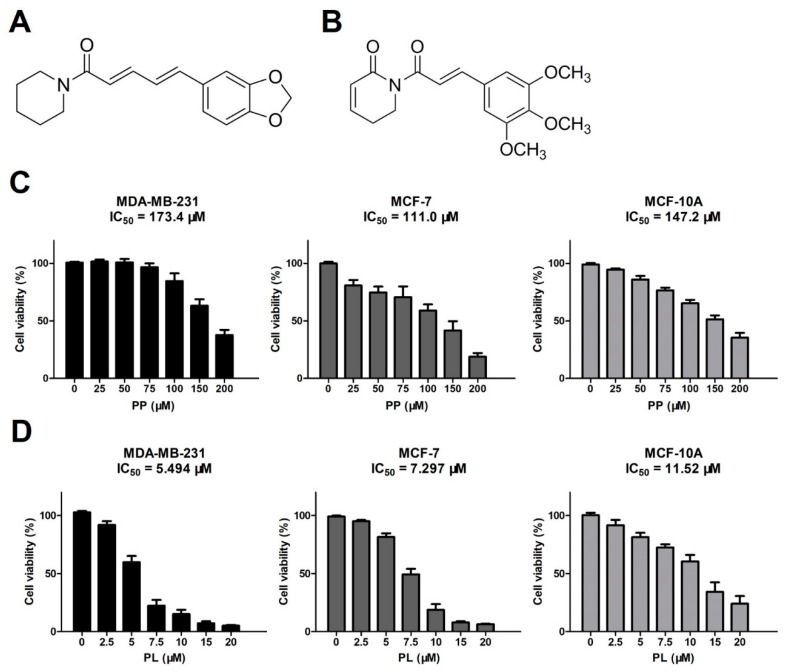
Piperine (PP) and piperlongumine (PL) inhibit the proliferation of breast cancer cells and normal breast cells. (**A**,**B**) Molecular structures of piperine and piperlongumine. (**C**,**D**) MDA-MB-231, MCF-7, and MCF-10A cells were treated with the indicated concentrations of PP and PL for 48 h, DMSO was used as a vehicle control, and cell viability was detected by MTT assay.

Recent studies have reported that both PP (Figure 1A) and PL (Figure 1B) have a broad spectrum of anticancer effects. We initially evaluated anti-proliferative activity of PP and PL against three human breast cell lines, including a triple-negative breast cancer (TNBC) cell line (MDA-MB-231), an ER/PR positive breast cancer cell line (MCF-7), and a normal cell line (MCF-10A).

All three cell lines were exposed to various concentrations of PP and PL for 48 h and were examined by MTT assay. As shown in Figure 1C, PP inhibited cell growth in a dose-dependent manner, with IC_50_ values of 173.4 μM (MDA-MB-231), 111.0 μM (MCF-7), and 147.2 μM (MCF-10A), respectively (Figure 1C). Similarly, PL inhibited the growth of all cell lines in a dose-dependent manner, however, showed stronger anti-proliferative activity, with IC_50_ values of 5.494 μM (MDA-MB-231), 7.297 μM (MCF-7), and 11.52 μM (MCF-10A), respectively (Figure 1D). The results suggest that PP has a weak inhibitory effect on breast cancer cells and normal cells, while PL effectively inhibits breast cancer cell proliferation and exhibits some selectivity in normal cells.

### 2.2. Piperine and Piperlongumine Induce Apoptosis in Breast Cancer Cells and Normal Breast Cells

Flow cytometry assay was then performed to study apoptotic induction effect of PP and PL. Three cell lines were treated with the indicated concentrations of PP and PL for 48 h, stained with Annexin V-FITC/PI, and then analyzed.

**Figure 2:**
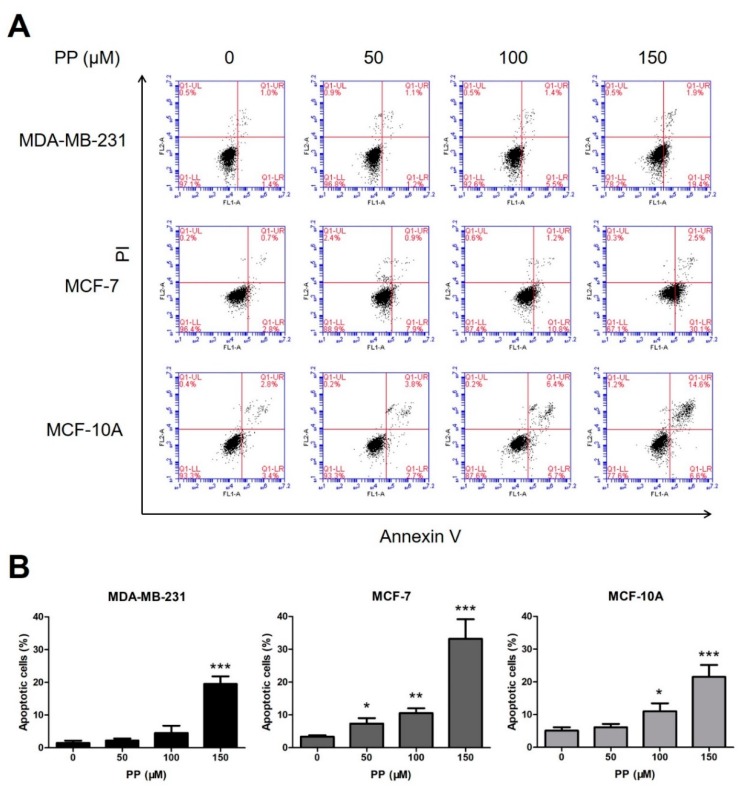

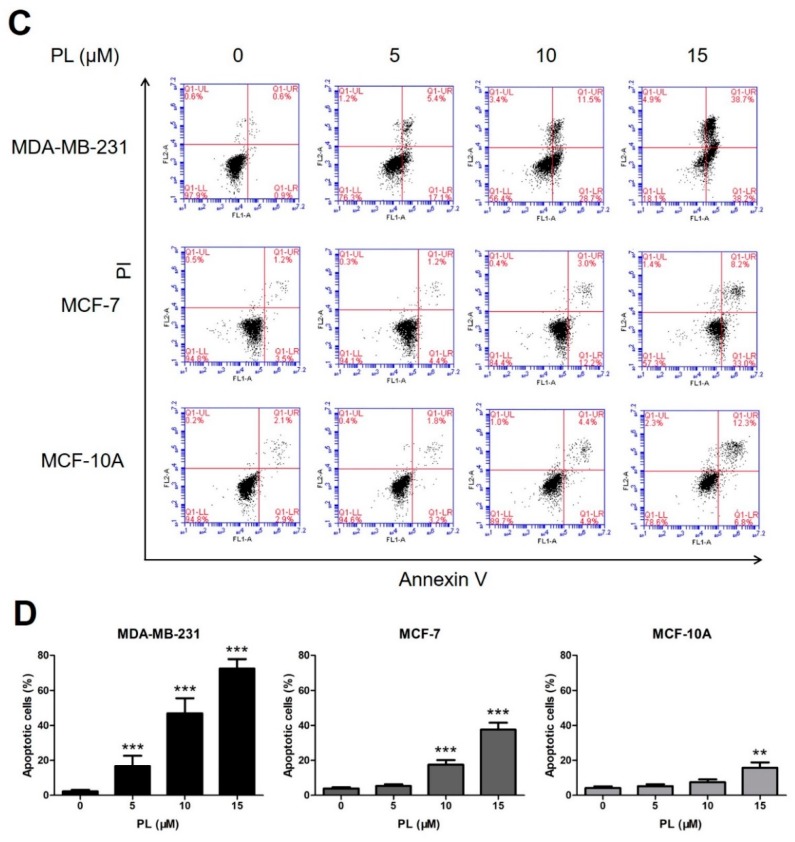
Piperine and piperlongumine induce apoptosis in breast cancer cells and normal breast cells. (**A**,**C**) MDA-MB-231, MCF-7, and MCF-10A cells were treated with the indicated concentrations of PP and PL for 48 h and stained with Annexin V/PI. DMSO was used as a vehicle control. The apoptotic rate was then detected by flow cytometry assay. (**B**,**D**) The percentage of apoptotic cells in the treatment groups was calculated. * *p* < 0.05, ** *p* < 0.01, *** *p* < 0.001 compared to the control group.

As shown in Figure 2A,B, PP induced apoptosis of MCF-7 cells at all doses but only induced apoptosis at highest dose in MDA-MB-231 cells, indicating that MDA-MB-231 is less sensitive to PP than MCF-7. As shown in Figure 2C,D, PL exhibited much stronger apoptotic induction activity, even though only one-tenth of the doses were tested compared to PP. Besides, PL showed certain selectivity in normal cells, which is consistent with previous MTT results.

### 2.3. Piperine and Piperlongumine Synergistically Inhibit the Proliferation of Breast Cancer Cells

**Figure 3:**
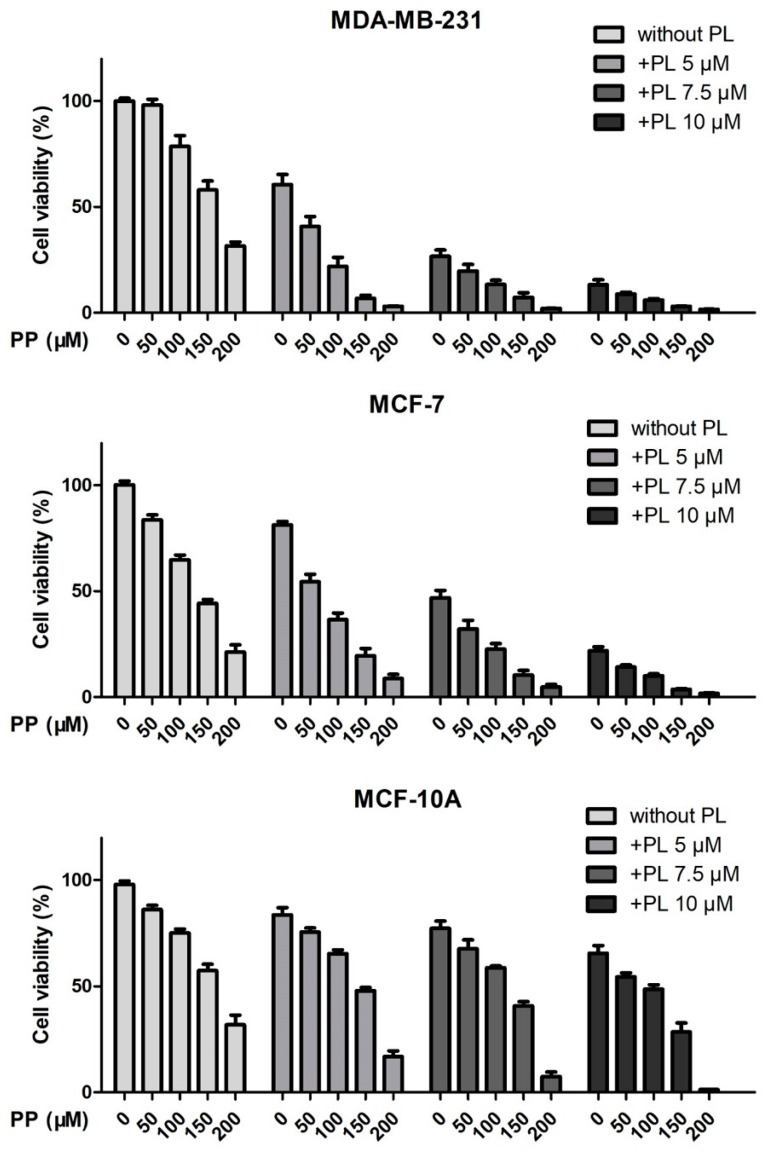
Piperine and piperlongumine synergistically inhibit the proliferation of breast cancer cells. MDA-MB-231, MCF-7, and MCF-10A cells were treated with the indicated concentrations of PP and PL for 72 h. DMSO was used as a vehicle control, and cell viability was detected by MTT assay.

Previous results demonstrated that PP showed weak inhibitory activity in breast cancer cells, particularly in TNBC, while PL exhibited good anticancer potential and selectivity. Therefore, we combined these two chemicals to evaluate whether there was a synergistic effect. MDA-MB-231, MCF-7, and MCF-10A cells were treated with PP (50 μM, 100 μM, 150 μM, 200 μM) and PL (5 μM, 7.5 μM, 10 μM) alone or in combination for 72 h, and then detected by MTT assay according to Chou’s design of drug combination studies (Figure 3).

Since these raw data could not explain the effect of drug combination on their own, they were brought into the CompuSyn software to obtain combination index (CI) values. CI < 1, =1, and >1 were considered to be synergism, additive effect, and antagonism. As shown in Table 1, CI values of PP 50 μM plus PL 5 μM and PP 100 μM plus PL 5 μM were 0.70 and 0.88 in MDA-MB-231 cells, while 0.76 and 0.92 in MCF-7 cells. However, CI values of above combination were greater than 1 in MCF-10A normal cells. The results indicate that the combination of PP and PL at lower doses exhibited a selective synergistic effect.

### 2.4. The Combination of Piperine and Piperlongumine Significantly Induces Apoptosis in Breast Cancer Cells

To further confirm previous data, we performed flow cytometry assay with combined PP and PL at lower doses. MDA-MB-231, MCF-7, and MCF-10A cells were treated with PP (50 μM, 100 μM) and PL (5 μM) alone or in combination for 48 h, stained with Annexin V-FITC/PI, and then analyzed.

**Figure 4:**
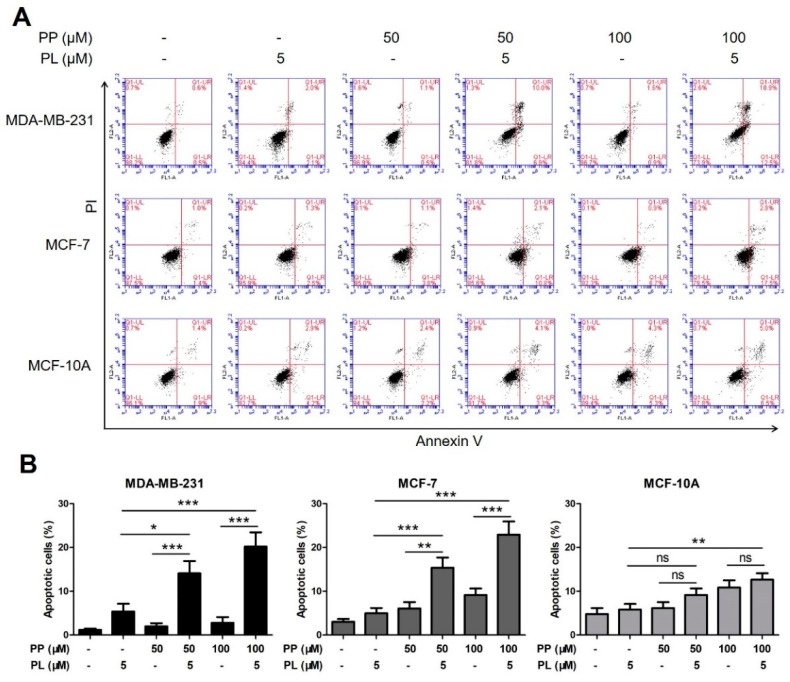
The combination of piperine and piperlongumine significantly induces apoptosis in breast cancer cells. (**A**) MDA-MB-231, MCF-7, and MCF-10A cells were treated with PP (50 μM, 100 μM) and PL (5 μM) alone or in combination for 48 h and then stained with Annexin V/PI. DMSO was used as a vehicle control, and the apoptotic rate was detected by flow cytometry assay. (**B**) The percentage of apoptotic cells in the treatment groups was calculated. * *p* < 0.05, ** *p* < 0.01, *** *p* < 0.001 compared to the compared group; ns: Not significant.

As shown in Figure 4A,B, the cell apoptotic rate was significantly increased by combination group compared with either agent alone. This remarkable effect was observed in both breast cancer cells but not in normal cells, indicating a good selectivity.

### 2.5. The Combination of Piperine and Piperlongumine Down-Regulates Bcl-2 in Breast Cancer Cells

To better understand the role of combination in apoptotic induction, protein levels of apoptosis-associated markers, Bcl-2 and Bax, were detected by Western blot assay. MDA-MB-231, MCF-7, and MCF-10A cells were treated with PP (50 μM, 100 μM) and PL (5 μM) alone or in combination for 24 h, levels of Bcl-2 and Bax were then analyzed.

**Figure 5:**
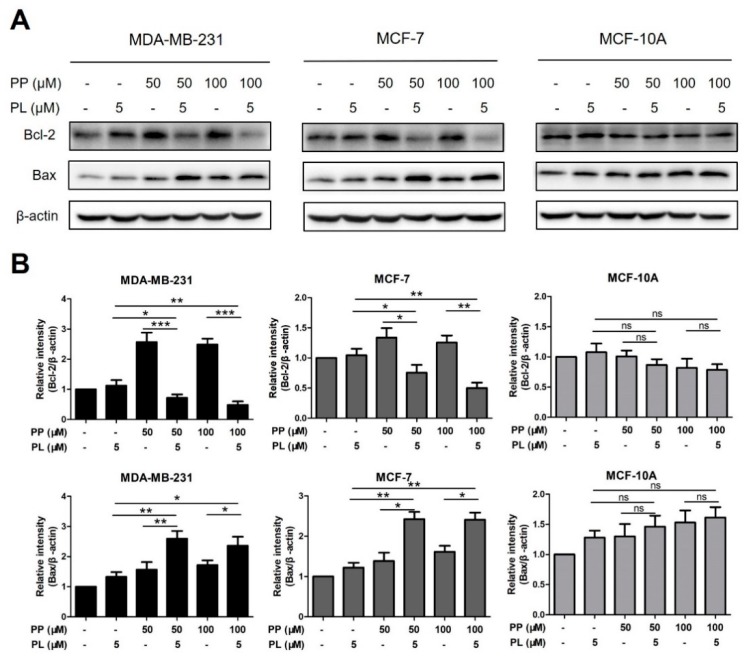
The combination of piperine and piperlongumine down-regulates Bcl-2 in breast cancer cells. (**A**) MDA-MB-231, MCF-7, and MCF-10A cells were treated with PP (50, 100 μM) and PL (5 μM) alone or in combination for 24 h. Cell lysates were then subjected to Western blot assay. Proteins levels of Bcl-2 and Bax were detected. β-actin was used as an internal control. (**B**) The relative protein expression in each treatment group was calculated. * *p* < 0.05, ** *p* < 0.01, *** *p* < 0.001 compared to the compared group; ns: Not significant.

As shown in Figure 5A,B, PP alone increased Bcl-2 levels in MDA-MB-231 cells, however, this effect was reversed when treating PP and PL together, indicating that PL may play a key role in downregulating Bcl-2. Upregulation of Bax by the combination treatment was also observed in two breast cancer cells. In addition, PP combined with PL did not alter any change of Bcl-2 or Bax in MCF-10A cells, which is consistent with previous apoptosis data.

### 2.6. The Combination of Piperine and Piperlongumine Inhibits STAT3 Phosphorylation in Breast Cancer Cells

To uncover the mechanism of combination treatment, protein levels of STAT3 pathway-related markers, including p-STAT3, STAT3, p-JAK2, JAK2, and survivin, were detected by Western blot assay. MDA-MB-231, MCF-7, and MCF-10A cells were treated with PP (50 μM, 100 μM) and PL (5 μM) alone or in combination for 24 h, and were then analyzed.

**Figure 6:**
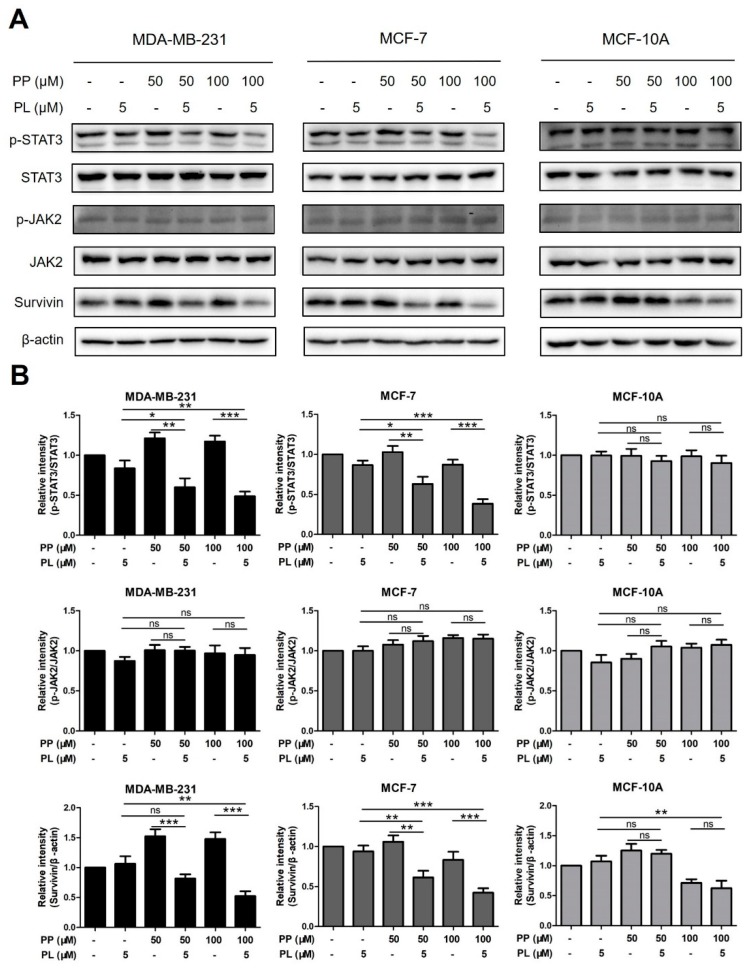
The combination of piperine and piperlongumine inhibits STAT3 phosphorylation in breast cancer cells. (**A**) MDA-MB-231, MCF-7, and MCF-10A cells were treated with PP (50 μM, 100 μM) and PL (5 μM) alone or in combination for 24 h. Cell lysates were then subjected to Western blot assay. STAT3 pathway-related proteins, including p-STAT3, STAT3, p-JAK2, JAK2, and survivin, were detected. β-actin was used as an internal control. (**B**) The relative protein expression in each treatment group was calculated. * *p* < 0.05, ** *p* < 0.01, *** *p* < 0.001 compared to the compared group; ns: Not significant.

As shown in Figure 6A,B, combination groups significantly reduced the levels of p-STAT3 in two cancer cell lines compared with either chemical alone. p-STAT3 levels were increased by PP alone in MDA-MB-231 cells, however, it could be reversed when treating PP and PL together, which was similar to previous Bcl-2 data. Moreover, the effect of combination on the downstream molecule survivin was consistent with p-STAT3, while no significant change was observed on the phosphorylation of JAK2, an upstream molecule of the STAT3 pathway. In addition, consistent with previous data, combination treatment did not alter any change in STAT3 pathway-related markers in MCF-10A normal cells. These results indicate that combination of PP and PL inhibits STAT3 phosphorylation in breast cancer cells, which may be a key mechanism for the selective killing.

## 3. Discussion

Pepper has been used as food and medicine for thousands of years worldwide. PP, the main component of pepper, has recently been reported to inhibit growth in different types of cancer cells [6,12,32,33]. Preclinical research indicated that PP has potential to become an anticancer agent [34]. Our data confirmed previous findings, that PP has weak anti-proliferative as well as apoptotic induction activity in two breast cancer cell lines (MDA-MB-231, MCF-7) and one normal breast cell line (MCF-10A). Poor killing activity and selectivity make it necessary to find another agent to sensitize breast cancer cells to PP.

PL is another natural alkaloid isolated from pepper, but shows stronger anticancer potential and better selectivity [14,35]. Unlike PP, the natural yield of PL is extremely low, and its synthesis route is neither simple nor environment friendly. In addition, poor water solubility and organ toxicity were observed at higher doses. However, previous studies have reported that PL can synergize with gemcitabine and doxorubicin in vitro and in vivo through NF-κB- and STAT3-mediated apoptosis [28,36], making PL an expected supplementary.

Considering the complementary advantages of PP and PL, we wonder whether combination treatment could achieve a better effect. Different doses of PP and PL were combined and tested by MTT assay according to Chou’s method [37]. CI analysis indicated that combination at medium and higher doses did not have an ideal synergistic killing effect, while synergistic effect at lower doses (PP 50 μM plus PL 5 μM, PP 100 μM plus PL 5 μM) were observed in both breast cancer cells but not in normal cells, indicating a selective synergistic effect. Similarly, the combination of PP and PL at lower doses induced a significant apoptotic effect compared with either agent in breast cancer cells but not in normal cells (Figure 4B). Consistent data demonstrated that the combination of these two chemicals exhibited more potent anticancer potential and satisfactory selectivity, which confirmed our speculation. However, the mechanism of apoptosis induced by combination treatment remains unclear.

Apoptosis is a strictly controlled pattern of programmed cell death which is essential for normal cell growth and plays a critical role in cancer therapy. It is regulated by several protein families, such as the Bcl-2 family which is a central regulator of caspase activation, including both pro-apoptotic proteins and anti-apoptotic proteins [38]. The opposing role of different members of this family, such as Bcl-2 and Bax, determine cell fate [39]. It has been reported that PL induces apoptosis of various cancer cells by downregulating Bcl-2 in a dose-dependent manner [16]. PP was also reported to decrease Bcl-2 levels in lung cancer and cervical cancer cells [40]. Based on the similar regulatory mechanisms, we analyzed the expression levels of Bcl-2 and Bax in all three cell lines by Western blot assay with single or combined treatment of PP and PL (Figure 5A,B). After 24 h, the regulatory effect of a single treatment was less obvious, probably due to a weaker effect at lower doses. Moreover, the combination of PP and PL significantly increased Bax levels while reduced Bcl-2 levels in MDA-MB-231 and MCF-7 cells, but no such effect was observed in MCF-10A cells. Surprisingly, both 50 μM and 100 μM PP increased Bcl-2 levels in MDA-MB-231 cells, which has not been reported before and may be one of the reasons why MDA-MB-231 cells are less sensitive to PP. Nevertheless, this unexpected effect can be reversed, as demonstrated by downregulated Bcl-2 after combination with PL, indicating that PL plays a key role in this combination system. Consistent data from Western blot and flow cytometry assays demonstrated that PL enhanced PP-induced apoptosis via regulation of Bcl-2-associated proteins and selectively killed breast cancer cells. The upstream molecular mechanism leading to this unique activity needs further exploration.

STAT3 signaling pathway plays a crucial role in proliferation (Bcl-2, Survivin), angiogenesis (HIF1α, VEGF), and epithelial-mesenchymal transition (Vimentin, MMP-9) in breast cancer cells and has been validated as a drug target for cancer therapy [41,42,43]. Several agents have been proved to induce apoptosis by inhibiting the STAT3 pathway, such as chelerythrine, sanguinarine and 4-chlorobenzoyl berbamine [44,45,46]. PL was identified as a direct STAT3 inhibitor with potent activity against breast cancer [47], while the role of PP to STAT3 regulation in breast cancer has not yet been reported. Based on our previous data, we speculated that the combination of these two chemicals may regulate downstream proteins and induce apoptosis through STAT3 pathway. For a better understanding, we analyzed the expression levels of STAT3 pathway-related proteins in all three cells with single or combined treatment of PP and PL (Figure 6A,B). After 24 h, the levels of p-STAT3, STAT3, p-JAK2, JAK2, and survivin were detected by Western blot assay. Single or combination groups did not appear to alter the relative expression of p-JAK2/JAK2 in any cell, indicating JAK2 may not be the target of this combination. However, the combination of PP and PL significantly suppressed STAT3 phosphorylation and survivin in MDA-MB-231 and MCF-7 cells, while no such effect was observed in MCF-10A cells.

Interestingly, PP not only failed to inhibit STAT3 activation but also increased levels of p-STAT3 as well as its downstream molecules survivin and Bcl-2 in MDA-MB-231 cells. We believe that mutational status of p53 could be responsible for these unexpected results. In the present study, both MCF-7 and MCF-10A cells have wild-type p53, whereas MDA-MB-231 cells do not. Moreover, it was previously reported that PP suppress Bcl-2 signaling pathway via inactivating STAT3 in p53 wild-type Hela cells [40], thus supporting our hypothesis. Mutant p53 loses its regulation of reducing STAT3 tyrosine phosphorylation and inhibiting DNA binding activity, causing cancer cells to escape PP-mediated killing effects [48]. Nevertheless, this unexpected effect can be reversed when treating PP and PL in combination. This result is highly consistent with Bcl-2 data and our previous report on PL reversal of DOX upregulation [36]. These findings indicate that PL is involved in increasing the sensitivity of MDA-MB-231 cells to PP. One explanation is that PP and PL complement their respective advantages, in which PL inactivates STAT3 through direct binding while PP improves the bioavailability of PL [49]. Then, the two chemicals together suppress STAT3 pathway-associated molecules and enhance apoptotic effect in MDA-MB-231 and MCF-7 cells. In addition, PL induces apoptotic cell death through increasing reactive oxygen species (ROS) levels in cells with cancer genotype, irrespective of p53 status [35], thereby leading a weak killing effect in MCF-10A normal cells. Taken together, blocking of STAT3 activation by combination of PP and PL was proven to be a mechanism of selective killing of breast cancer cells.

This study reports for the first time that PL enhances PP-induced apoptosis and selectively kills breast cancer cells. Data demonstrated that inhibition of STAT3 activation could be a novel mechanism against MDA-MB-231 and MCF-7 cells, which is dependent on neither hormone type nor p53 status. The combination of PP and PL at lower doses exhibits potent anticancer potential as well as good selectivity, which provides a new strategy for the prevention and complementary therapy of breast cancer.

## 4. Materials and Methods

### 4.1. Chemicals

Piperine and piperlongumine were purchased from ApexBio (Houston, TX, USA). Both agents were dissolved in dimethyl sulfoxide (DMSO) and diluted in culture medium.

### 4.2. Cell Culture

The breast cancer cell lines MDA-MB-231, MCF-7, and MCF-10A were purchased from American Type Culture Collection (ATCC, Manassas, VA, USA). Cells were cultured in Dulbecco’s Modified Eagle’s Medium (DMEM, Thermo Fisher Scientific, Waltham, MA, USA), supplemented with 10% FBS (Thermo Fisher Scientific, Waltham, MA, USA) and 100 U/mL penicillin and streptomycin (Thermo Fisher Scientific, Waltham, MA, USA). Cells were cultured with 5% CO_2_ in a 37 °C incubator.

### 4.3. Cell Viability Assay

Cells were seeded at 3–5 × 10^3^ cells/well in 96-well plates with overnight incubation and exposed to certain concentrations of PP and PL, alone or in combination, for 48 h of incubation. Then an MTT working solution was added, and the plate was further incubated for 4 h. Subsequently, the medium was discarded and 100 μL of DMSO was added to each well to dissolve the formazan crystals. The absorbance in each well was measured at 570 nm using an Epoch microplate reader (BioTek, Winooski, VT, USA). The combination index (CI) values were analyzed using Chou-Talalay method and were calculated using CompuSyn software (Version 1.0, ComboSyn, Inc., New York, NY, USA). CI <1, =1, and >1 were considered to be synergism, additive effect, and antagonism in drug combinations.

### 4.4. Cell Apoptosis Analysis

Cells were seeded in 6-well plates for overnight incubation and then treated with PP and PL, alone or in combination, for 48 h of incubation. Cells were then harvested, washed twice with ice-cold PBS, and double stained with FITC-conjugated Annexin V and PI in binding buffer for 15 min. The rate of apoptosis was evaluated using an Accuri C6 flow cytometer (BD, Franklin Lakes, NJ, USA).

### 4.5. Western Blot Analysis

Cells were homogenized in protein lysate buffer and collected after centrifugation at 12,000× *g* for 10 min at 4 °C. The protein concentrations were determined by BCA protein assay kit (Beyotime, Shanghai, China). Then total proteins with equal amounts were subjected to sodium dodecyl sulfate-polyacrylamide gel electrophoresis (SDS-PAGE) and electro-transferred onto poly-vinylidene difluoride (PVDF) membranes. The blots were blocked for 1 h at room temperature with 5% nonfat milk and then incubated with specific primary and secondary antibodies. The β-actin (Beyotime, Shanghai, China) was used as an internal control. The primary antibodies included Bcl-2, Bax, p-JAK2, JAK2, p-STAT3, STAT3 and survivin (Cell Signaling Technology, Danvers, MA, USA).

### 4.6. Statistical Analysis

The results of all tests expressed as the mean ± SD of three independent experiments. All statistical analyses were conducted using GraphPad Prism software (Version 5.0, GraphPad Software Inc., La Jolla, CA, USA). Statistical significance between multiple treatment groups was analyzed using one-way ANOVA. *p* < 0.05 was considered statistically significant. 

## Figures and Tables

**Table 1:** CI values of piperine and piperlongumine with combination treatment in MDA-MB-231, MCF-7, and MCF-10A cells.

Cell Lines	Combined Treatments	CI Values
MDA-MB-231	PP50 + PL5	0.70
	PP100 + PL5	0.88
	PP150 + PL5	1.09
	PP200 + PL5	1.40
	PP50 + PL7.5	0.82
	PP100 + PL7.5	1.39
	PP150 + PL7.5	1.65
	PP200 + PL7.5	1.38
	PP50 + PL10	0.96
	PP100 + PL10	1.47
	PP150 + PL10	1.53
	PP200 + PL10	1.66
MCF-7	PP50 + PL5	0.76
	PP100 + PL5	0.92
	PP150 + PL5	1.08
	PP200 + PL5	1.42
	PP50 + PL7.5	1.03
	PP100 + PL7.5	1.69
	PP150 + PL7.5	1.89
	PP200 + PL7.5	1.40
	PP50 + PL10	1.13
	PP100 + PL10	1.77
	PP150 + PL10	1.86
	PP200 + PL10	1.70
MCF-10A	PP50 + PL5	1.35
	PP100 + PL5	1.68
	PP150 + PL5	1.69
	PP200 + PL5	0.90
	PP50 + PL7.5	1.04
	PP100 + PL7.5	1.64
	PP150 + PL7.5	1.68
	PP200 + PL7.5	0.75
	PP50 + PL10	0.79
	PP100 + PL10	1.34
	PP150 + PL10	1.06
	P200 + PL10	0.51
